# Ergebnisse der systematischen Literatursuche als Grundlage für die „evidenzbasierten Therapieempfehlungen für FMF-Patienten mit unzureichendem Ansprechen bzw. Unverträglichkeit auf Kolchizin“ der Gesellschaft für Kinder- und Jugendrheumatologie und der Deutschen Gesellschaft für Rheumatologie

**DOI:** 10.1007/s00393-020-00886-0

**Published:** 2020-09-30

**Authors:** T. Sahr, U. Kiltz, C. Weseloh, T. Kallinich, J. Braun

**Affiliations:** 1 Rheumazentrum Ruhrgebiet, Herne, Ruhr-Universität Bochum, Claudiusstr. 45, 44649 Herne, Deutschland; 2 Deutsche Gesellschaft für Rheumatologie, Berlin, Deutschland; 3 Pädiatrie m.S. Pulmonologie und Immunologie, Sozialpädiatrisches Zentrum, Universitätsmedizin Charité Berlin, Berlin, Deutschland

**Keywords:** Familiäres Mittelmeerfieber, Biologika, Kolchizin-Resistenz, Europäische Liga gegen den Rheumatismus, Interleukin-1, Familial Mediterranean fever, Biologics, Colchicine resistance, European League Against Rheumatism, Interleukin 1

## Abstract

**Hintergrund:**

Das familiäre Mittelmeerfieber (FMF) ist eine in Deutschland eher seltene genetisch bedingte Erkrankung des Kindes- und Erwachsenenalters, die durch rezidivierende Fieberschübe sowie Peritonitis, Pleuritis und Arthritis charakterisiert ist. Die etablierte Therapie mit Kolchizin ist für die meisten Patienten wirksam und verträglich. Einige Patienten sprechen aber auf diese Therapie nicht ausreichend an bzw. vertragen diese nicht. Für diese Patienten kommen Biologika in Betracht. Die Gesellschaft für Kinder- und Jugendrheumatologie (GKJR) und die Deutsche Gesellschaft für Rheumatologie (DGRh) sind übereingekommen, gemeinsame Empfehlungen für diese spezielle klinische Situation zu entwickeln.

**Ziel:**

Durchführung einer systematischen Literaturrecherche (SLR) auf Basis der 2016 publizierten EULAR(European League Against Rheumatism)-Empfehlungen als Grundlage für die Entwicklung von evidenzbasierten Therapieempfehlungen für FMF-Patienten mit unzureichendem Ansprechen bzw. Unverträglichkeit auf Kolchizin.

**Methoden:**

Die SLR wurde mit Referenzen aus verschiedenen Datenbanken und als Aktualisierung der bis zum Jahr 2014 durchgeführten SLR der EULAR durchgeführt, wobei die Artikel zwischen dem 01.01.2015 und dem 31.12.2017 publiziert worden sein mussten. Für die Vorselektion wurde das Abstractwerkzeug Rayyan und für die Erstellung der Evidenztabellen die Klassifikation des Oxford Centre for Evidence Based Medicine 2009 benutzt.

**Ergebnisse:**

Die Suche ergab 360, nach Dublettenabgleich noch 263 Treffer. Insgesamt 88 Publikationen wurden ein- (34%) und 102 ausgeschlossen (39%), bei weiteren 73 war eine Sichtung der Vollpublikation notwendig (28%), und 43 wurden intensiver diskutiert. Schlussendlich blieben 64 Publikationen (24%) übrig. Insgesamt wurden 4 Fall-Kontroll-Studien, 31 Kohortenstudien, 8 Fallserien, 7 kontrollierte Studien (davon 5 Abstracts), 10 Übersichtsarbeiten sowie 4 Metaanalysen und systematische Reviews akzeptiert.

**Diskussion:**

Die SLR wurde wissenschaftlich exakt, transparent und nach internationalen Standards durchgeführt. Die SLR erwies sich als gute Grundlage für die Konsentierung der 5 übergeordneten Prinzipien und der 10 Empfehlungen, sodass die gemeinsame Aktivität von GKJR und DGRh erfolgreich und sogar zeitnah abgeschlossen werden konnte. Die Empfehlungen sind eine solide Basis, Patienten jeden Alters mit FMF gut zu behandeln. Dabei spielen die Erklärungen zum Problem der Kolchizinresistenz eine wichtige Rolle.

**Zusatzmaterial online:**

Die Online-Version dieses Beitrags (10.1007/s00393-020-00886-0) enthält die Evidenztabellen, Tabellen zum Verzerrungsrisiko (RoB) sowie die Übersicht der Suchstrategien. Folgende Inhalte finden Sie online: Tab. 2a: Evidenztabelle für alle zitierten Fall-Kontroll-Studien, Studiencharakteristika; Tab. 2b: Evidenztabelle für alle zitierten Kohortenstudien, Studiencharakteristika; Tab. 2c: Evidenztabelle für alle zitierten Fallserien, Studiencharakteristika; Tab. 2d: Evidenztabelle für sämtliche zitierte kontrollierte Studien (inklusive Abstracts), Studiencharakteristika; Tab. 2e: Evidenztabelle für alle zitierten Übersichtsarbeiten, Studiencharakteristika; Tab. 2f: Evidenztabelle für alle zitierten Metaanalysen und systematischen Reviews, Studiencharakteristika; Tab. 3a: Verzerrungsrisiko für sämtliche zitierte Kohortenstudien nach der Newcastle-Ottawa-Skala (Risiko für Bias); Tab. 3b: Verzerrungsrisiko für sämtliche zitierte Fall-Kontroll-Studien nach der Newcastle-Ottawa-Skala (Risiko für Bias); Suchstrategien (Supplement). Beitrag und Zusatzmaterial stehen Ihnen auf www.springermedizin.de zur Verfügung. Bitte geben Sie dort den Beitragstitel in die Suche ein, das Zusatzmaterial finden Sie beim Beitrag unter „Ergänzende Inhalte“.

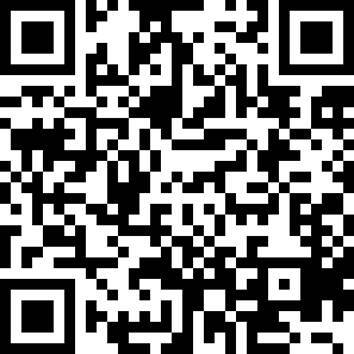

## Abstract (en)

**Background:**

Familial Mediterranean fever (FMF) is a genetic disease of childhood and adulthood which is relatively rare in Germany. It is characterized by recurrent febrile attacks, peritonitis, pleuritis and arthritis. The established treatment with colchicine is effective and well-tolerated by most patients; however, some patients do not adequately respond or do not tolerate this treatment. Biologics can be considered for some of these patients. The Society for Pediatric and Adolescent Rheumatology (GKJR) and the German Society for Rheumatology (DGRh) have agreed to develop joint recommendations for this specific clinical situation.

**Aim:**

Implementation of a systematic literature search (SLR) on the basis of the EULAR recommendations published in 2016 as the foundation for the development of evidence-based treatment recommendations for FMF patients with insufficient response or intolerance to colchicine.

**Methods:**

The SLR was performed using references from various databases as an update of the SLR carried out by EULAR up to 2014, whereby all articles must have been published between 1 January 2015 and 31 December 2017. The Rayyan abstract tool for the preselection and the classification of the Oxford Centre for Evidence Based Medicine 2009 were used for the preparation of the evidence tables.

**Results:**

The search yielded 360 hits and after duplicate matching 263. A total of 88 publications were included (34%) and 102 excluded (39%), a review of the full publication was necessary for a further 73 (28%) and 43 were discussed more intensively. Finally, 64 publications (24%) remained. A total of 4 case-control studies, 31 cohort studies, 8 case series, 7 controlled studies (including 5 abstracts), 10 reviews, 4 meta-analyses and systematic reviews were accepted.

**Discussion:**

The SLR was carried out in a scientifically accurate and transparent manner according to international standards. The SLR proved to be a good basis for a consensus on the 5 overarching principles and the 10 recommendations, so that the joint activity of the GKJR and DGRh was successfully and even promptly concluded. The recommendations are a solid basis for treating patients of all ages with FMF. The explanations on the problem of colchicine resistance play an important role here.

Das familiäre Mittelmeerfieber (FMF) ist eine autoinflammatorische Erkrankung, die durch rezidivierende Fieberschübe, häufig in Kombination mit klinischen Zeichen einer Peritonitis, Pleuritis und/oder Arthritis, charakterisiert ist [1]. Bei unbehandelten oder unzureichend behandelten Patienten erhöht die häufig zum Teil subklinische Entzündungsreaktion das Risiko für das Auftreten von Komplikationen, wie z. B. Amyloidose, erheblich [2]. Klassifikationskriterien für das FMF sind 1997 vorgeschlagen worden [3].

Ätiopathogenetisch ist die Erkrankung mit Mutationen im *ME*diterranean-*FE*ver-Gen (*MEFV*) assoziiert, wobei aber kein klassischer Erbgang vorliegt, denn es handelt sich um eine Gendosis-Wirkungs-Beziehung [4–10]. Vorschläge für eine rationale Durchführung genetischer Diagnostik sind 2012 gemacht worden [11].

Ziel des FMF-Managements ist es, FMF-Attacken und mögliche Organkomplikationen zu verhindern [2]. Die pharmakologische Therapie basiert im Wesentlichen auf Kolchizin. Die prophylaktische und therapeutische Wirksamkeit des Mitosehemmstoffs Kolchizin, welches aus der ziemlich giftigen Herbstzeitlosen (*Colchicum autumnale*) gewonnen wird, ist seit fast 50 Jahren etabliert [12–17].

Die klinische Wirksamkeit dieser Therapie wurde schon vor vielen Jahren nachgewiesen, als gezeigt wurde, dass klinisch apparente Attacken von FMF meist komplett und bei vielen Patienten partiell verhindert werden konnten [12–17]. Die kontinuierliche Therapie mit Kolchizin stellte auch einen wirksamen Schutz vor Amyloidose dar [12]. Evidenzbasierte Empfehlungen für den Einsatz von Kolchizin bei Patienten mit FMF mit detaillierter Angabe von zugelassenen Dosierungen wurden früh publiziert [16, 17] und sind auch Teil der European League against rheumatism (EULAR)-Empfehlungen von 2016 [18]. Da in der wissenschaftlichen Literatur der Begriff „Kolchizin-Resistenz“ nicht genau definiert ist bzw. unterschiedliche Definitionen publiziert wurden und bisher keine internationale Übereinstimmung erzielt werden konnte, ist davon auszugehen, dass das Management bei Patienten mit „Kolchizin-resistentem familiärem Mittelmeerfieber“ uneinheitlich erfolgt ist. Eine Übersicht über die bisher gemachten Vorschläge gibt Tab. 1.

**Table 1:** 

Referenz	Jahr	Definition der Kolchizin-Resistenz
Barut, K. et al. [33]	2017	≥1 Attacke/Monat über 3 Monate
Basaran, Ö. et al. [34]	2015	Schwere, häufige Attacken und/oder erhöhte Entzündungsparameter
Ben-Zvi, I. et al. [25]	2017	≥1 Attacke/Monat
Cetin, P. et al. [35]	2015	Nicht eindeutig (schwere, gehäufte Attacken oder erhöhte Entzündungsparameter trotz Maximaldosis)
De Benedetti, F. et al. [36]	2017	≥1 Attacke/Monat
Eroglu, F.K. et al. [37]	2015	≥1 Attacke in den letzten 3 Monaten und Erhöhung von Entzündungsparametern zwischen den Attacken
Knieper, M. et al. [38]	2017	<6 Attacken/Jahr oder >3 Attacken in den letzten 4 bis 6 Monaten (3,7 % der Gesamtkohorte)
Kucuksahin, O. et al. [39]	2016	≥2 Attacken/Monat und Erhöhung von CRP und BSG zwischen den Attacken
Laskari, K. et al. [40]	2017	Aktive Erkrankung trotz mindestens 3 Monate langer Kolchizin-Therapie
Lofty, H.M. et al. [41]	2016	≥1 Attacke/Monat
Melikoglu, M.A. et al. [42]	2017	≥1 Attacke alle 3 Monate
Omma, A. et al. [43]	2017	>3 Attacken in den letzten 6 Monaten
Ozen, S. et al. [44]	2017	≥1 Attacke/Monat über 3 Monate
Ozer, I. et al. [45]	2015	≥3 Attacken in 6 Monaten
Ozkan, S. et al. [46]	2017	Schwere, häufige Attacken und/oder erhöhte Entzündungsparameter
Pecher, A.C. et al. [47]	2017	>1 Attacke/Monat und/oder >1,5-facher Anstieg der Attackenfrequenz im Vergleich zum Zeitpunkt des besten Ansprechens auf eine Kolchizin-Therapie
Yalcintepe, S. et al. [48]	2016	≥2 Attacken/Monat und Erhöhung der Entzündungsparameter

Reicht die Therapie mit Kolchizin nicht aus bzw. wird diese nicht vertragen, können danach Biologika wie die IL-1-Inhibitoren eingesetzt werden [23]. Interleukin 1 (IL-1) ist ein wichtiger Vermittler von Entzündungsreaktionen, der bei den natürlichen Reaktionen des Körpers und der Entwicklung pathologischer Zustände, die zu chronischen Entzündungen führen, eine wichtige Rolle spielt [19]. Durch eine unangemessen hohe Produktion und Freisetzung von IL-1β kann eine Vielzahl unterschiedlicher Symptome wie Arthritis, Exantheme, Konjunktivitis, Serositis, Fieber oder Hörverlust ausgelöst werden [20]. Die Verwendung von IL-1-Antagonisten hat sich bei Patienten mit autoinflammatorischen Zuständen, die mit einer übermäßigen IL-1-Produktion einhergehen, als wirksam erwiesen, die erste Publikation dazu gab es 2007 [21]. Die Implikation von IL‑1 in den durch pathogene Kristalle ausgelösten Entzündungsprozess lieferte die Begründung für die Verwendung von IL-1-Inhibitoren z. B. bei kristallinduzierter Arthritis [22, 23].

Nachdem erst nur Fallberichte und kleine Kohortenstudien veröffentlicht worden waren, die den erfolgreichen Einsatz einer IL-1-Blockade bei Patienten mit unzureichendem Ansprechen auf Kolchizin beschrieben, gab es dann zunehmend auch randomisierte kontrollierte Studien zum Einsatz der IL-1-Antagonisten Rilonacept [24], Anakinra [25] und zuletzt Canakinumab [26] bei Patienten mit hoher Krankheitsaktivität eines FMF trotz adäquat dosierter Kolchizin-Therapie.

Seit 2017 ist Canakinumab für die Therapie des FMF in Deutschland zugelassen. Weil auf der einen Seite nur relativ wenige Patienten für eine solche Therapie infrage kommen und auf der anderen Seite insbesondere die Behandlung mit Canakinumab mit relativ hohen Kosten verbunden ist sowie keine Daten zur Langzeittherapie bei großen Kohorten vorliegen, waren möglichst eindeutige Empfehlungen für den Einsatz von Biologika bei therapierefraktärem FMF wünschenswert. Im April 2020 hat die European Medical Association (EMA) im Rahmen einer Indikationserweiterung auch Anakinra für die Behandlung des FMF zugelassen.

Da im Jahr 2016 EULAR-Empfehlungen zum Management von FMF publiziert worden waren, wurden diese als Ausgangspunkt für die Erstellung nationaler Therapieempfehlungen gewählt. Ziel der vorliegenden Arbeit war es, unter Berücksichtigung der neuesten Studienergebnisse und der aktuellen Zulassungssituation in Deutschland die Evidenzbasis zu schaffen, um erweiterte Empfehlungen zur Therapie des FMF für betroffene Patienten und Ärzte, die mit dem Management von FMF-Patienten betraut sind, entwickeln und konsentieren zu können [27]. Dies betrifft vorwiegend Rheumatologen und Pädiater mit Erfahrung in der Behandlung solcher Patienten.

## Methoden

Wegen dieses Problems und angesichts der neuen Therapiemöglichkeiten haben die Gesellschaft für Kinder- und Jugendrheumatologie (GKJR) und die Deutsche Gesellschaft für Rheumatologie e. V. (DGRh) beschlossen, auf Basis einer systematischen Literaturrecherche (SLR) eine gemeinsame Empfehlung zur „Therapie des Kolchizin-resistenten familiären Mittelmeerfiebers“ zu erarbeiten.

Teilnehmer der Konsensusgruppe waren: Norbert Blank (DGRh), Jürgen Braun (DGRh, Koordinator), Eugen Feist (DGRh), Tilmann Kallinich (GKJR), Uta Kiltz (DGRH), Ulrich Neudorf (GKJR), Prasad Oommen (GKJR), Helmut Wittkowski (GKJR) und Christiane Weseloh (Wissenschaftliche Mitarbeiterin der DGRh). Der medizinische Doktorand Tom Sahr (geb. Braun) half bei der Erstellung der SLR, unterstützte die Volltextselektion und erstellte die Evidenztabellen. Um den Anforderungen einer evidenzbasierten Publikation gerecht zu werden, wurden die Interessenkonflikte der stimmberechtigten Mitglieder vor Projektbeginn von jedem Teilnehmer erhoben. Die finale Bewertung wurde von U. Kiltz, T. Kallinich und J. Braun vorgenommen.

### Methodik der Erstellung der Therapieempfehlungen

#### a) Durchführung der systematischen Literaturrecherche mit Suchstrategien (MEDLINE, Cochrane)

Für die Durchführung der SLR wurden vorab der Suchzeitraum, die Datenbanken, Publikationsart und das Suchschema mittels PICO-Fragen von der Gruppe konsentiert. PICO steht als Akronym für:

*P*opulation oder Patient, Problem: Beschreibung der Gruppe von Patienten bzw. des Problems.

*I*ntervention = Technologien, diagnostisches/therapeutisches Verfahren: Welche Intervention ist Gegenstand der gegenwärtigen Untersuchung?

*C*omparison oder Control = Vergleichsintervention: Was ist die Hauptalternative, mit der die Intervention verglichen werden kann?

*O*utcome = Zielgröße: Was soll erreicht werden?

Die SLR sollte unter Verwendung von Referenzen aus MEDLINE-, EMBASE- und Cochrane CENTRAL-Datenbanken und als Aktualisierung der bis zum Jahr 2014 durchgeführten SLR der EULAR durchgeführt werden [18, 28, 29]. Die in der vorliegenden SLR enthaltenen Artikel mussten zwischen dem 01.01.2015 und dem 31.12.2017 publiziert worden sein.

Die gefundenen Artikel wurden zunächst nach Titel und Abstract gemäß den Ein- und Ausschlusskriterien gesichtet. Duplikate wurden manuell aussortiert. Die Referenzen wurden in ein Abstractverwaltungstool (Rayyan) hochgeladen und vorselektiert. Die vorselektierten Abstracts standen den Arbeitsgruppen zur weiteren Selektion zur Verfügung.

Die Volltextorganisation der relevanten Arbeiten wurde in der Universitätsmedizin Charité Berlin arbeitsteilig durchgeführt. In einem weiteren Schritt selektierten die Mitglieder der Konsensusgruppe teamweise diejenigen Arbeiten, die für die Publikation von Interesse waren und formal und inhaltlich den Vorgaben entsprachen.

**Figure 1:**
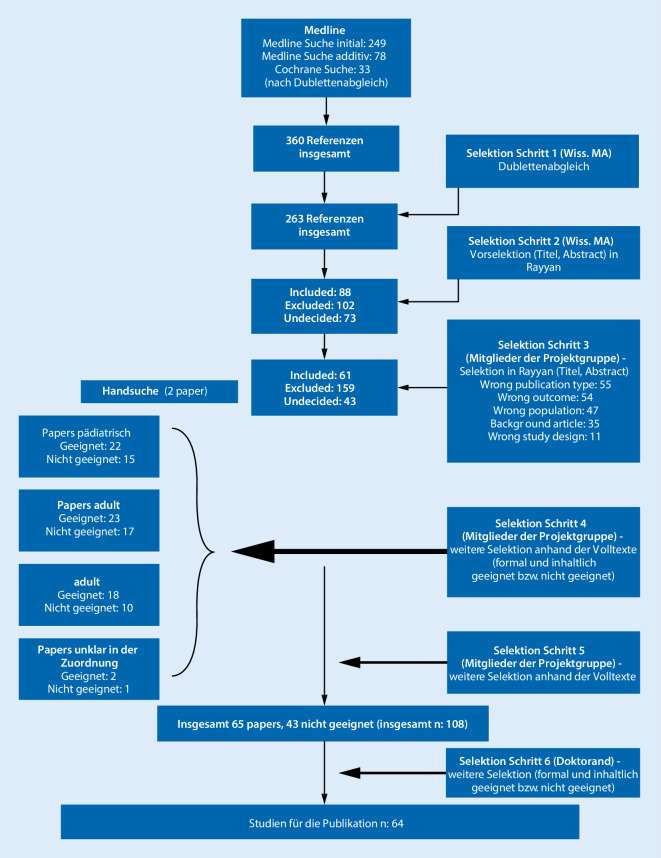

Die Gruppe wurde bei der Selektion vom Doktoranden unterstützt, der am Ende des Auswahlprozesses zudem eine umfassende Evidenzbewertung vornahm. Dabei wurde eine weitere Selektion der Studien anhand der formalen und inhaltlichen Eignung vorgenommen (s. Abb. 1).

#### b) Formulierung der Therapieempfehlung

Die Formulierung der Therapieempfehlungen basierte auf der identifizierten Evidenz und wurde von der Gruppe auf Basis eines offenen Austausches formuliert. Für die Verabschiedung der Empfehlungen vereinbarte die Gruppe, dass eine Mehrheit von 75 % zur Annahme eines Formulierungsvorschlages notwendig sein sollte.

## Ergebnisse

### A) Literatursuche

#### Durchführung:

Die SLR wurde am 28.01.2018 in den Datenbanken MEDLINE (PubMed) und Cochrane Library auf Basis der PICO-Fragen für den Zeitraum 01.01.2015 bis 31.12.2017 durchgeführt (s. Zusatzmaterial online):

P (Population): alle Patienten mit familiärem Mittelmeerfieber (FMF),

I (Intervention): alle Medikamente, die eingesetzt wurden, wenn Kolchizin nicht ausreichte,

C (Comparator): Glukokortikoide und konventionelle disease modifying anti-rheumatic drugs (DMARDs),

O (Outcome): Krankheitsaktivität inklusive C-reaktivem Protein (CRP), S‑100 und Serum-Amyloid‑A, Amyloidose.

Als Selektionskriterien wurden im Vorfeld die Publikationsarten wie folgt definiert:

- Reviews,
- Editorials,
- randomisierte kontrollierte Studien (RCT), (10 Patienten und mehr, Laufzeit ≥12 Wochen),
- Kohorte (25 Patienten und mehr, Laufzeit 6 Monate und mehr, wenn Längsschnittarm),
- Fall-Kontroll-Studie (20 Patienten und mehr, Laufzeit 6 Monate und mehr, wenn Längsschnittarm),
- Metaanalyse,
- Case Reports (mindestens 3 Patienten):
  - Arbeit in Englisch oder Deutsch,
  - Volltextpublikation + Kongressabstracts.

Ausschlusskriterien:

- Zweitpublikation
- Gruppen nicht sauber getrennt (z. B. FMF und andere periodische Fiebersyndrome vermischt)

Eine Intervention war nicht zwingend vorgeschrieben.

#### Datenextraktion und Bewertung des Verzerrungspotenzials.

Jeder identifizierte Artikel oder Abstract wurde unabhängig von je 2 Reviewern auf Eignung gemäß den vordefinierten Einschlusskriterien bewertet, gefolgt von einer Volltextüberprüfung. Für jede eingeschlossene Studie wurden relevante Daten extrahiert und in Evidenztabellen zusammengefasst (**Tab. 2a–f**). Die Bewertung der Evidenz erfolgte mithilfe der Oxford Center of Evidence-based Medicine-Levels of Evidence von 2009 (www.cebm.net). (s. Zusatzmaterial online). Darüber hinaus bewerteten die Gutachter das Verzerrungspotenzial (risk of bias, RoB) jeder Studie anhand des „Cochrane-Tools“ für RCTs, des „Hayden-Tools“ für Kohortenstudien und der Newcastle-Ottawa-Skala für den Einzelfall [28–30]. Meinungsverschiedenheiten hinsichtlich der Eignung der Studien, der Datenextraktion und der RoB-Bewertung wurden durch Diskussion und Konsens gelöst. Bei anhaltenden Meinungsverschiedenheiten wurde ein dritter Gutachter hinzugezogen.

Die Suche ergab insgesamt 360 Treffer (nach Dublettenabgleich: 263 Treffer). Die Referenzen wurden in ein Abstractverwaltungstool (Rayyan) hochgeladen und vorselektiert (eingeschlossen: 88, ausgeschlossen: 102, keine sichere Zuordnung: 73). Die vorselektierten Abstracts standen den Arbeitsgruppen zur weiteren „Selektion“ zur Verfügung.

Die Abstractselektion durch die Mitglieder der Arbeitsgruppen ergab zum Stichtag 01.03.2018 folgendes Ergebnis: eingeschlossen: 61, ausgeschlossen: 159, keine sichere Zuordnung: 43. In einem weiteren Schritt selektierten die Mitglieder der Konsensusgruppe teamweise diejenigen Arbeiten, die für die Publikation von Interesse waren und formal und inhaltlich den Vorgaben entsprachen.

Am Ende des Auswahlprozesses wurde eine umfassende Evidenzbewertung vorgenommen mit weiterer Selektion der Studien anhand der formalen und inhaltlichen Eignung. Schlussendlich blieben *64* Publikationen übrig, die für die Empfehlungen zu berücksichtigen waren. Insgesamt wurden 4 Fall-Kontroll-Studien, 31 Kohortenstudien, 8 Fallserien, 7 kontrollierte Studien (davon 5 Abstracts), 10 Übersichtsarbeiten und 4 Metaanalysen und systematische Reviews als Grundlage für die Formulierung der Therapieempfehlung identifiziert (Abb. 1).

Die Evidenztabellen und die RoB-Bewertungsliste finden Sie als Zusatzmaterial online.

### B) Konsentierung der übergeordneten Prinzipien und Empfehlungen

Auf einem Projekttreffen im Rheumazentrum Ruhrgebiet in Herne am 19.04.2018 wurde auf Basis der gefundenen Evidenz die Sachlage in der Gruppe diskutiert, und übergeordnete Prinzipien und Empfehlungen wurden konsentiert. Es waren 7 stimmberechtigte Teilnehmer der Gruppe anwesend, keiner fehlte. Das Ergebnis dieser Konsentierung wurde den Vorständen von DGRh und GKJR zur Prüfung und Freigabe vorgelegt. Änderungswünsche wurden diskutiert und partiell berücksichtigt. Diese Änderungen wurden erneut mittels einer elektronischen Abfrage zwischen den Autoren konsentiert. Die Kommission Pharmakotherapie der DGRh (Sprecher: Klaus Krüger) war in den Freigabeprozess der Publikation ebenso eingebunden. Sowohl die übergeordneten Prinzipien als auch die Empfehlungen basieren auf den EULAR-Empfehlungen und einer aktualisierten SLR. Es wurden 5 übergeordnete Prinzipien und 10 Empfehlungen formuliert. Alle Empfehlungen wurden nach intensiver inhaltlicher Diskussion in der Expertengruppe in voller Übereinstimmung (100 %) konsentiert [27].

## Diskussion

Die SLR war insgesamt trotz des relativ engen Zeitraums (im Anschluss an die EULAR-Empfehlungen [23] von 2016) formal, quantitativ (Referenzen [28, 31–89]), inhaltlich und auch qualitativ erfolgreich (Abb. 1; Tab. 1**–2a–f**). Die gemeinsame Arbeitsgruppe von DGRh und GKJR hat dann auch auf dieser Basis sehr gut funktioniert, sodass die gemeinsamen Empfehlungen schon im Jahr 2019 publiziert werden konnten [27]. Neben den hier ausführlich vorgestellten Publikationen wurden für die Empfehlungen noch einige ältere Publikationen für die Themen Kolchizin inklusive Dosierung und Resistenz sowie Sicherheit [92–102], Lebensqualität [103–107] und Schwere der Erkrankung [108–112], Akutphaseparameter [113–115], Arthritis [116–119] febrile Myalgien [120–122] und Nebenwirkungen bzw. Applikationsform von Biologika [123–125] hinzugezogen.

Interessanterweise haben sich die möglichen Indikationen für Kolchizin in Richtung Perikarditis [126, 127] und Canakinumab in Richtung Arteriosklerose [128, 129] und sogar Myokardinfarkt [130] inzwischen ausgeweitet.

Ein Ende 2017 für die Behandlung von Gicht und FMF von der EMA neu zugelassenes Kolchizin-haltiges Arzneimittel wird in Deutschland inzwischen flächendeckend vertrieben.

Nachdem der erfolgreiche Einsatz von IL-1-Antagonisten bei FMF-Patienten mit unzureichendem Ansprechen auf Kolchizin zunächst nur in Fallberichten und kleinen Kohortenstudien beschrieben worden war, gibt es inzwischen eine ziemlich solide Datenlage für die Wirksamkeit der 3 IL-1-Antagonisten, wobei nur Anakinra und Canakinumab in Deutschland erhältlich sind. So liegen für den Einsatz von Rilonacept, Anakinra und Canakinumab bei Patienten mit hoher Krankheitsaktivität eines FMF trotz adäquat dosierter Kolchizin-Therapie mittlerweile publizierte Ergebnisse randomisierter kontrollierter Studien vor. Für Canakinumab gibt es auch bereits recht positive 72-Wochen-Daten [131].

Inwieweit auch andere Biologika wirksam sind, kann noch nicht abschließend beurteilt werden, für anti-IL-6R und anti-TNF‑α gibt es aber bislang immer noch lediglich positive Fallberichte [132].

Interessant geblieben ist auch das Thema Assoziationen und Komorbidität, hierzu wurde kürzlich eine große Studie vorgelegt, in der auch Zusammenhänge zwischen FMF und Spondyloarthritiden bzw. Morbus Behçet wieder thematisiert bzw. mit Daten gestützt wurden [133].

## Caption Electronic Supplementary Material


