# Supplementary material for: Results of the systematic literature search as basis for the “Evidence-based treatment recommendations for familial Mediterranean fever patients with insufficient response or intolerability to colchicine” of the Society for Pediatric and Adolescent Rheumatology and the German Society for Rheumatology
Source: Z Rheumatol. 2020 Sep 30;79(9):943–51. [Article in German] doi: 10.1007/s00393-020-00886-0 (PMC7647992; doi:10.1007/s00393-020-00886-0)
Supplement: Supplementary file 1 [file 393_2020_886_MOESM1_ESM.pdf]

Tabelle 2a: Evidenztabelle für alle zitierten Fall-Kontroll-Studien, Studiencharakteristika

Autor	Jahr	Population	Kontrolle	Ziel / Intervention	Ergebnisse	Kommentar	Evidenz-stärke
Ben-Zvi, I et al. (9)	2015	164 Patienten mit FMF, davon 47 ohne Mutationen im MEFV-Gen (zum Alter k.A.)	60 heterogene Kontrollen, 57 p.M694V - homozygote Kontrollen	Klinische Ausprägung bei Patienten ohne Mutationen im MEFV-Gen	FMF-Patienten ohne Nachweis einer Mutation haben einen leichteren Verlauf mit weniger betroffenen Körperstellen, weniger Attacken und weniger verordneten Colchizin-Dosen.	Interessant, klinische Relevanz aber unklar.	3b
Brenner R et al. (49)	2018	n = 8.534 Patienten mit FMF (Alter 43.74 Jahre)	Israelische Bevölkerung	Tumorinzidenz bei Patienten mit FMF	Im Vergleich zur Gesamt-bevölkerung weisen Patienten mit FMF eine geringere Tumorinzidenz auf.	Dies kann an physiologischen Merkmalen der Erkrankung oder an der Behandlung liegen.	3b
Migita K et al. (50)	2015	64 Patienten, davon 20 mit FMF (mittleres Alter 34 ± 7.8 Jahre)	22 gesunde Kontrollen, 22 Patienten mit rheumatoider Arthritis	Analyse der Rolle von zirkulierendem gespaltenen IL-1 β (p17)	Ratio von IL-1 β (p17): leichte Kette der Immunglobuline erhöht bei Patienten mit FMF im Vergleich zu Patienten mit rheumatoider Arthritis (0.064 vs. 0.01).	Die gespaltene Form von IL-1 β ist ein möglicher Biomarker für das Monitoring von Krankheitsaktivität und das Ansprechen auf eine Colchizin-Therapie.	3b
Yalcintepe S et al. (48)	2016	90 FMF-Patienten, 60 mit Colchizin-Resistenz (zum Alter k.A.)	30 FMF-Patienten mit Ansprechen auf Colchizin	Assoziation von CYP2D6 Allelen mit Nicht-Ansprechen auf die Colchizin-Therapie	Die Prävalenz des CYP2D6 *4 und *6 Allels ist bei Patienten mit Nicht-Ansprechen auf Colchizin erhöht.	Interessant, klinische Relevanz aber unklar.	2b

Tabelle 2b: Evidenztabelle für alle zitierten Kohortenstudien, Studiencharakteristika

Autor	Jahr	Populationen	Fokus/Intervention	Ergebnisse	Kommentar	Evidenzgrad
Akar S et al. (51)	2016	172 Patienten mit FMF, 84% mit Colchizin-Resistenz, 12% mit Amyloidose (mittleres Alter 36 Jahre)	Effektivität einer IL-1-Blockade mit Anakinra (n=151) und Canakinumab (n=21)	Reduktion der Attackenfrequenz von 16.8/Jahr auf 2.4/Jahr ($p < 0.001$). Insgesamt 42.1% der Patienten mit Colchizinresistenz waren asymptomatisch.	Eine anti-IL-1-Therapie ist eine effektive Alternative, um FMF-Attacken zu kontrollieren und um bei Colchizin-resistenten FMF-Patienten die Proteinurie zu vermindern.	2c
Barut K. et al. (33)	2017	708 Patienten mit FMF (Alter 12.3 ± 4.4 Jahre)	Effektivität einer Therapie mit Colchizin bzw. mit IL-1-Blockern: Anakinra (n=4), Canakinumab (n=12)	Unter Colchizin-Therapie 79% der Patienten ohne Attacken, 14.4% partielles Ansprechen, 6.6% Colchizin-Resistenz. Gutes Ansprechen einer IL-1-Blockade bei allen damit behandelten Patienten.	Bestätigte Wirkung von Colchizin. Vielversprechende Daten mit IL-1-Blockern bei Colchizin-resistentem FMF.	4
Bayram MT et al. (52)	2015	105 Kinder mit FMF, davon 20% mit subklinischer Inflammation (100% <18 Jahre)	Assoziation von klinischen, demographischen und genetischen Faktoren mit dem Ausmaß der subklinischen Entzündung	Myalgien und Erysipel-artige Hautläsionen sind unabhängige Risikofaktoren für das Vorliegen einer subklinischen Entzündung.	Um Langzeitschäden besser verhindern zu können, sind größere Studien erforderlich, um Risikofaktoren für subklinische Entzündungen und deren Auswirkungen besser einschätzen zu können.	4

Bonfrate L et al. (53)	2017	49 Patienten mit FMF (mittleres Alter 38 Jahre [5 – 69 Jahre])	Klinische und genetische Beschreibung von FMF-Patienten unter Colchizin	> 95% deutliche Verbesserung nach Einleitung einer Colchizin-Therapie	Colchizin ist und bleibt der Goldstandard in der Therapie des FMF.	4
Bonyadi MJ et al. (54)	2015	415 Patienten mit FMF (mittleres Alter 26 Jahre, zwischen 3 und 78 Jahren)	Klinische und genetische Beschreibung von FMF-Patienten	95% der Patienten verbessern sich nach Einleitung einer Colchizin-Therapie.	Colchizin ist und bleibt der Goldstandard in der Therapie des FMF.	2b
Cetin P et al. (35)	2015	20 Patienten mit FMF (mittleres Alter 23 Jahre [15 – 50])	Effektivität einer IL-1-Blockade mit Anakinra (n=12), Canakinumab (n=8)	Reduktion der Attackenfrequenz von 15/Jahr auf 0.5/Jahr ($p = 0.001$).	Offene Studie, anti-IL1-Therapie scheint bei Colchizin-resistentem FMF wirksam zu sein.	4
Corsia A et al. (55)	2017	51 Patienten mit FMF, alle mit Colchizinresistenz (mittleres Alter 34 Jahre, zwischen 4 und 86 Jahren)	Beschreibung von Kriterien, die das Vorliegen einer Colchizin-Resistenz nahelegen.	Komplette Medikamenten-Adhärenz wurde von 40% der Patienten (48% Kinder, 22% Erwachsene) bestätigt. Die behandelnden Ärzte nahmen eine Colchizin-Resistenz bei Vorliegen der folgenden Konstellationen an: > 6 Attacken/Jahr (42%), > 4 Attacken in letzten 4 Monaten (26%), anhaltende Inflammation (45%), renale Amyloidose (22%), und Auftreten von Nebenwirkungen bei Colchizin-Dosiserhöhung (19%).	Geringe Compliance bei der Colchizin-Einnahme scheint eine wichtige Komponente beim Vorliegen von Colchizin-Resistenz zu sein und sollte daher vor Diagnosestellung evaluiert werden.	2b
Dermikaya E et al. (56)	2015	281 Patienten mit FMF (162 Kinder, 119 Erwachsene)	Entwicklung eines Scores zur Einschätzung der Krankheitsschwere bei FMF	Nach eigenen Angaben interne Validität demonstriert.	Eine Validierung sollte erfolgen, um die Einsatzfähigkeit zu bestätigen.	2b

Dogan H et al. (57)	2015	1.620 Patienten mit Verdacht auf FMF (mittleres Alter 16 Jahre [1–72])	Identifikation von seltenen Mutationen	- Identifikation von 731 Patienten mit Mutationen<br>- Identifikation von 27 symptomatischen Patienten mit seltenen Mutationen	Kein Erkenntnisgewinn für die Behandlung von Colchizin-resistentem FMF, Daten fügen sich in das Gesamtbild.	4
Erden A et al. (58)	2018	385 FMF-Patienten wurden evaluiert und Patienten, die eine Anti-Interleukin-1-Behandlung erhielten, einbezogen.	11 verschiedene Definitionen (nach der PubMed-Suche nach Colchicin-Resistenz in FMF gefunden) wurden auf alle Patienten angewendet. Die Ergebnisse wurden erneut analysiert, nachdem die Patienten, die keine klinischen Attacken, aber anhaltend hohe Akute-Phase-Proteine und/oder Amyloidose hatten, ausgeschlossen wurden.	60 Patienten (40 Erwachsene/20 Kinder), die eine Anti-IL-1-Therapie angewendet hatten, wurden als Colchizin-resistente Patienten in diese Studie einbezogen. Der höchste Prozentsatz der Patienten erfüllte Definition 5 (93,3%). Definition 9 hatte die geringste Übereinstimmung (26%). Bezeichnenderweise erfüllte ein höherer Prozentsatz der erwachsenen Patienten die Definitionen 4 und 6 als pädiatrische Patienten (87,5% vs. 50%, p=0,002; 75% vs. 40%, p=0,008). Nach dem Ausschluss von Patienten ohne klinische Attacken erfüllte der höchste Prozentsatz der Patienten Definition 2 (94,4%). Man kombinierte die Attackenhäufigkeit (>1 typische Episode/3 Monate) in Definition 2 und das Vorhandensein von Amyloidose/Akute-Phase-Protein-Erhöhung in Definition 5, um eine neue Definition zu schaffen, die von 59 (98,3%) Colchizin-resistenten FMF-Patienten erfüllt wurde.	Die Definition von Colchicin-Resistenz ist nach wie vor umstritten. Definitionen mit klinischen und Laborkriterien wurden von einem höheren Prozentsatz resistenter Patienten erfüllt als solche ohne Laborkriterien. Die richtigen Definitionen für den attackenfreien Zeitraum und die Persistenz von Entzündungsmarkern fehlen jedoch noch.	2b

Eroglu K et al. (37)	2015	14 Patienten mit Colchizin-resistentem FMF, chronischer Arthritis und protrahierten febrilen Myalgien)<br>(mittleres Alter 13,2 Jahre, zwischen 2 und 24 Jahren)	Effektivität einer anti-TNFα-Therapie mit Etanercept (n=3), und einer anti-IL-1-Therapie mit Anakinra (n=11), und Canakinumab (n=9)	Etanercept waren bei einem Patienten erfolglos, bei einem Patienten führte es zu einer Neutropenie, ein Patient mit chronischer Arthritis hatte eine partielle Verbesserung. Unter Anakinra zeigten alle Patienten (2 nach Dosiserhöhung) zumindest zeitweilig eine klinische Verbesserung. Alle Patienten wiesen unter Canakinumab eine Verbesserung auf, wobei Dosen und Intervalle variierten. Am Ende des Beobachtungszeitraums hatten 2 Patienten weiterhin klinische Symptome und erhöhte Entzündungsparameter.	Kleine offene Studie, suggeriert eine ausreichende Wirksamkeit IL1-Blockern bei Colchizin-resistentem FMF, aber nicht bei allen Patienten.	2b
Herscovici T et al. (59)	2015	37 mit Colchizin behandelte Mütter (38 exponierte Kinder) und 75 mit nicht embryotoxischen Medikamenten behandelte Mütter als Kontrollgruppe (76 exponierte Kinder)	Sicherheit der Colchizintherapie während der Stillzeit (n=37)	Keine Unterschiede in der Häufigkeit von Nebenwirkungen bei Colchizin-exponierten Kindern.	Colchizin scheint während der Stillzeit weiter gegeben werden zu können.	**3b**

Konukbay D et al. (60)	2016	250 Kinder mit FMF wurden in die Studie einbezogen.	Der multidimensionale Beurteilungsbericht für jugendliche autoentzündliche Erkrankungen (JAIMAR) umfasst 16 eltern- oder patientenzentrierte Maße und vier Punkte, die den Funktionsstatus, Schmerzen, die therapeutische Compliance und die gesundheitsbezogene Lebensqualität (physisch, sozial, schulisch, emotional) mit Outcome bewerten. Es wird vorgeschlagen, sowohl als Proxy-Bericht als auch als Patientenselbstbericht zu verwenden, bei 8-18 jährigen Patienten	Es gab eine signifikante und eine positive Korrelation zwischen den Test-Retest-Scores (ICC=0.607-0.966). Hinsichtlich der Validität des Aufbaus lagen alle Faktoren über 0,30. Für die Validität des Kriteriums reichte die Korrelationsebene zwischen den einzelnen Punkten und der zugehörigen Skala von mittel (r=0,329, p-0,0001) bis groß (r=0,894, p-0,0001). Die von den Eltern gemeldeten und die selbst gemeldeten Daten der Kinder waren stark übereinstimmend (r=0.770-0.989)	Mit der Entwicklung des JAIMAR wurde ein neuer und mehrdimensionaler Ansatz in der pädiatrischen Rheumatologiepraxis eingeführt. Es ist ein neues Werkzeug für Kinder mit auto-inflammatorischen Erkrankungen und es kann dazu beitragen, ihre Qualität der Behandlung zu verbessern	**2b**

Knieper M et al. (38)	2017	409 Patienten mit FMF (100% <18 Jahre)	Assoziation von Faktoren, welche die Colchizin-Dosierung beeinflussen. Identifikation von Parametern, die eine Erhöhung der Colchizin-Dosis vorhersagen.	Körperoberfläche ist das anthropometrische Maß, das am besten mit der applizierten Colchizin-Dosis korreliert. Eine Kombination von Biomarkern unter Berücksichtigung der S100-Konzentration Moleküle kann eine Dosiserhöhung des Colchizins mit hoher Wahrscheinlichkeit vorhersagen. Die Einführung einer Colchizintherapie sowie eine spätere Dosissteigerung führt zu einem deutlichen Rückgang der Entzündungsmarker sowie der Attackenfrequenz.	Colchizin bleibt der Goldstandard in der Therapie des FMF.	2b
Kucuksahin O et al. (39)	2016	26 FMF-Patienten, davon 20 mit Colchizin-Resistenz und 8 mit Colchizin-Unverträglichkeit, sowie 3 mit persistierender Arthritis (Alter 22 - 57)	Effektivität einer IL-1-Blockade mit Anakinra (n=24) und Canakinumab (n=2)	20 Patienten blieben symptomfrei, 6 hatten weniger Attacken.	Kleine, offene Studie, vielversprechende Ergebnisse.	4

Laskari K et al. (40)	2017	14 Patienten mit Colchizin-resistentem FMF (mittleres Alter 38,5 Jahre [13-70 Jahre])	Effektivität einer IL-1-Blockade mit Canakinumab (n=14)	Unter Canakinumab erreichten 79% eine vollständige und 21% eine partielle Remission.	Behandlung mit Canakinumab in einem individuellen Dosierungs-schema resultierte in schneller und bleibender Remission bei Colchizin-resistentem FMF.	4
Lofty HM et al. (41)	2016	71 Patienten mit FMF (mittleres Alter 9 Jahre)	Assoziation des Serum-Amyloid A (SAA)-Spiegels mit klinischer Präsentation, Genotyp und anderen Entzündungswerten unter Colchizin	Keine signifikante Korrelation erhöhter SAA-Werte mit demographischen Daten und klinischer Präsentation.	Kein Erkenntnisgewinn für die Behandlung von Colchizin-resistentem FMF, Daten fügen sich aber ins Gesamtbild.	2b
Melikoglu MA et al. (42)	2017	108 Patienten mit FMF (mittleres Alter 42 Jahre)	Anzahl der Patienten mit Colchizin-Resistenz (n=10) und Beschreibung der (sozio-) demografischen und klinischen Aspekte	11% nahmen Colchizin unregelmäßig ein und 5% erfüllten die Definition für eine Colchizin-Resistenz. Unregelmäßige Colchizin-Einnahme ist mit geringerem sozioökonomischen Status und Bildungsniveau sowie Arbeitslosigkeit assoziiert.	Vor Diagnosestellung einer Colchizin-Resistenz sollten die Umstände der Colchizin-Einnahme und die Gewohnheiten der Patienten kritisch evaluiert werden.	2b
Omma A et al. (43)	2017	178 Patienten, davon 118 mit FMF und 60 gesunde Kontrollen (mittleres Alter jeweils ca. 36 Jahre)	Assoziation der nativen Thiol- und Disulfid-Spiegel mit Attacken und Colchizin-Resistenz unter Colchizin	Bei FMF-Patienten ist das native Thiol erniedrigt, insbesondere während der Attacken, zu diesem Zeitpunkt finden sich erhöhte Disulfid-Spiegel. Bei Patienten mit Colchizin-Resistenz lassen sich erhöhte Disulfid-Spiegel nachweisen.	Kein Erkenntnisgewinn für die Behandlung von Colchizin-resistentem FMF, Daten fügen sich aber in das Gesamtbild.	2b

Özcakar Z et al. (61)	2016 4	13 Patienten mit FMF, 7 davon Colchizin-resistent, und 6 mit FMF-assoziierter Amyloidose (Alter 2 - 24 Jahre)	Effektivität einer IL-1-Blockade mit Anakinra (n=10), und Canakinumab (n=3)	Bei Patienten mit Colchizin-resistenz Reduktion der mittleren Attackenfrequenz von 36/Jahr auf 3/Jahr (p = 0.018). Bei Patienten mit Amyloidose sind nach Einführung der Biologikatherapie keine Attacken mehr aufgetreten.	Kleine offene Studie, suggeriert Wirksamkeit einer IL-1-Blockade bei Colchizin-resistentem FMF sowie FMF-assoziierter Amyloidose.	4
Özdel E et al. (62)	2016	317 Patienten mit FMF (mittleres Alter 12 Jahre [1.5-22 Jahre])	Klinische Ausprägung bei spätem Manifestationsalter von FMF	Bei Patienten mit später Manifestation (nach dem 8. Lebensjahr) zeigt sich ein milderer klinischer Verlauf und es lässt sich seltener eine homozygote M694V-Mutation nachweisen, Colchizin wird niedriger dosiert.	Kein Erkenntnisgewinn für die Behandlung von Colchizin-resistentem FMF, Daten fügen sich jedoch ins Gesamtbild.	4
Ozen S et al. (44)	2017	134 Patienten, davon 49 mit FMF (Alter 73% <18 Jahre)	Beschreibung von Diagnosefindung und Therapieeinleitung	Therapie der FMF-Patienten mit Anakinra (n=14), Canakinumab (n=8), TNF-Blockern (n=4), und Interferon-α (n=1). Unter Anakinra waren 50% und unter TNF-Blockern 25% symptomfrei.	Offene Studie, vielversprechende Ergebnisse.	4
Ozer I et al. (45)	2015	64 Patienten mit FMF, davon 29 mit Colchizin-Resistenz (mittleres Alter 32 ± 2.4 Jahre)	Assoziation des Vitamin D-Spiegels mit einer Colchizin-Resistenz	Mittlere 25-Hydroxy-Vitamin D-Konzentration: Patienten mit Colchizin-Resistenz 9.39 ± 1.00 ng/ml, Patienten ohne Colchizin-Resistenz 18.48 ± 1.09 ng/ml ($p < 0.001$), also signifikant höher.	Kein sicherer Erkenntnisgewinn für die Behandlung von Colchizin-resistentem FMF, möglicher Einfluss des Vitamin D-Spiegels.	4

Pecher AC et al. (47)	2017	13 Colchizin-resistente Patienten mit FMF (mittleres Alter 31 [19-49]	Effektivität einer IL-1-Blockade mit Anakinra (n=13)	5 Patienten erreichten komplette und 8 partielle Remission. Die Attackenfrequenz sank von im Mittel 12/Jahr auf 2/Jahr.	Kleine offene Studie, suggeriert Wirksamkeit von Anakinra bei Colchizin-resistentem FMF.	4
Proft F et al. (63)	2017	26 Patienten mit verschiedenen auto-inflammatorischen Erkrankungen, 6 davon FMF	Effektivität und Sicherheit des Einsatzes unterschiedlicher Biologika im off-label Einsatz bei auto-inflammatorischen Erkrankungen, davon 5 mit Anakinra	38,5% sehr gutes bis gutes Ansprechen, 30,8% mäßiges Ansprechen, 30,7% schlechtes Ansprechen.	Retrospektive, nicht-interventionelle, multizentrische Registerstudie.<br>Kleine Fallzahl für FMF, positive Tendenz.	3
Rossi-Semerano L et al. (64)	2015	189 verschiedene inflammatorische Erkrankungen, 14 mit FMF<br>(mittleres Alter 21 Jahre [6 – 61 Jahre]	Effektivität und Sicherheit einer IL-1-Blockade mit Anakinra (n=13) und Canakinumab (n=4)	47% komplettes, 47% partielles, 6% kein Ansprechen	Insgesamt vielversprechende Daten für Anakinra bei Colchizin-resistentem FMF, aber relativ geringe Fallzahl, offene Studie. Zu wenig Patienten mit Canakinumab behandelt	4
Stankovic Stojanovic K et al. (65)	2016	218 Patienten mit FMF (mittleres Alter 27 Jahre)	Ermittlung eines Schwellwerts für C-reaktives Protein (CRP), welcher mit einem SAA-Spiegel < 10 mg/l korreliert	Bei Kindern korreliert ein CRP-Wert ≤ 5 mg/l und bei Erwachsenen ein CRP-Wert ≤ 8.75 mg/l mit einem SAA-Wert < 10 mg/l.	Kein Erkenntnisgewinn für die Behandlung von Colchizin-resistentem FMF, Daten fügen sich jedoch in das Gesamtbild.	2b

Topaloglu R et al. (66)	2016	30 Kinder mit FMF, davon 1 mit Colchizin-Resistenz (alle <18 Jahre)	Klinische Ausprägung bei Vorliegen von E148Q-Mutationen	Klinische Ausprägung: 50% mild, 47% moderat, 3% schwer. Ansprechen einer Colchizintherapie: 73% komplett, 23% partiell, 3% kein Ansprechen.	Kein Erkenntnisgewinn für die Behandlung von Colchizin-resistentem FMF, Daten passen aber zum Gesamtbild.	**4**
Vitale A et al. (67)	2016	526 verschiedene inflammatorische Erkrankungen, 34 davon mit FMF<br>(Alter 42 ± 20 Jahre)	(i)Frequenz der Behandlung von Patienten mit IL1-blockierenden Substanzen,<br>(ii)Häufigkeit des Wechsels zwischen Anakinra und Canakinumab,<br>(iii)Effektivität nach Wechsel der Substanzen,<br>(iv)Effektivität einer Dosisanpassung,<br>(v)Nebenwirkungsprofil,<br>(vi)Gründe für Therapieabbruch<br>(vii)Anakinra (n=421) und Canakinumab (n=105)<br>Unklar, wie viele mit FMF dabei waren.	FMF-spezifische Auswertung nicht möglich.	Daten passen zum positiven Gesamtbild.	2b

Yesilkaya S et al. (68)	2015	115 Patienten mit FMF (mittleres Alter 11 Jahre)	Entwicklung eines Instruments zur Beurteilung der Colchizin-Adhärenz bei FMF	Es gab eine positive und signifikante Korrelation zwischen Test- und Re-Testergebnissen (r=0.843; p<0.001). Außerdem gab es eine signifikante Korrelation zwischen den Angaben von Eltern- und Kindern (r=0.781, p<0.001).	Validierter Fragebogen, der Anhand von Punktwerten eine Aussage über die Medikamenten- Adhärenz bei Colchizin erlaubt.	2b
Yoldas TC et al. (69)	2015	51 Patienten mit FMF (Alter 6.4 ± 2.39])	Effekt einer Colchizin-Therapie und der Krankheitsaktivität auf das Wachstum	Bei Patienten, bei denen die Symptome komplett durch Colchizin kontrolliert werden, kommt es zu einem vermehrten Längenwachstum und einer gesteigerten Gewichtszunahme. Dies wird bei Patienten mit anhaltenden klinischen Symptomen nicht beobachtet.	Colchizin bleibt der Goldstandard in der Therapie des FMF.	2b

Tabelle 2c: Evidenztabelle für alle zitierten Fallserien, Studiencharakteristika

Autor	Jahr	Populationen	Fokus/Intervention	Ergebnisse	Kommentar	Evidenzgrad
Alaygut D et al. (70)	2016	15 Patienten mit FMF und 2 mit M. Behçet oder Familienmitglieder von jungen Patienten, die wegen Verdacht auf Colchizin-Vergiftung untersucht wurden (mittleres Alter 71 [18 – 204] Monate)	Beschreibung vom klinischen Effekt, der Behandlung und dem Outcome von Colchizin-Intoxikationen.	13 Patienten nahmen Colchizin in subtoxischer Dosierung ein (< 0.5 mg/kg/Tag). 7 Patienten zeigten Symptome einer Intoxikation, alle Patienten erholten sich vollständig.	Es besteht die Möglichkeit von Colchizin-Intoxikationen aus verschiedenen Gründen.	4
Basaran Ö et al. (34)	2015	8 Colchizin-resistente Patienten mit FMF, (Alter 11 - 19 Jahre)	Effektivität einer IL-1-Blockade mit Anakinra (n=8), davon nach 6 Monaten 4 x Canakinumab	Bei 7 Patienten klinische Remission, 1 Patient zeigte eingeschränkte HWS-Beweglichkeit.	Anakinra und Canakinumab scheinen bei Colchizinresistenz effektiv zu sein. Kontrollierte Studien sind aber erforderlich.	4
Ozkan S et al. (46)	2017	4 Colchizin-resistente Patienten mit FMF, 3 davon Geschwister (Alter 5 - 16 Jahre)	Effektivität einer IL-1-Blockade mit Anakinra (n=4) und Canakinumab (n=4)	Bis auf einen Patienten unter Anakinra zeigte sich bei allen eine komplette klinische Remission.	Anakinra und Canakinumab scheinen bei Colchizin-Resistenz effektiv zu sein. Kontrollierte Studien sind erforderlich.	4
Rom E et al. (71)	2018	8 Patienten mit febrilen Myalgien, FMF (mittleres Alter 10 [6 – 17] Jahre)	Effektivität einer Steroid-Pulstherapie	Steroidpulstherapie ist bei protrahiert verlaufenden Myalgien effektiv, führt aber nicht zu einer kompletten Kontrolle der klinischen Symptomatik.	Offene Studie, Glukokortikoide sind bei FMF-Patienten mit febrilen Myalgien wahrscheinlich effektiv.	4

Venhoff N et al. (72)	2017	3 FMF-Patienten mit 4 Schwangerschaften	Sicherheit von Anakinra während der Schwangerschaft	In allen Fällen unauffällige frühkindliche Entwicklung. In einem Fall aufgrund erhöhtem Komplikationsrisiko primäre Sectio in der 33. Schwangerschafts-woche.	Zu kleine Fallzahlen für eine vernünftige Aussage.	4
Yahia SH et al. (73)	2017	6 Patienten mit FMF (Alter 24 - 71 Jahre)	Helicobacter pylori-Infektion behandelt mit Clarithromycin	Auftreten einer Clarithromycin-assoziierten Colchizin-Intoxikation bei niereninsuffizienten Patienten unter Standarddosierung mit Colchizin möglich	Es besteht die Möglichkeit einer Clarithromycin-assoziierten Colchizin-Intoxikation.	4
Yilmaz S et al. (74)	2015	11 Patienten mit FMF und sekundärer renaler Amyloidose, 8 davon mit nephrotischem Syndrom (Alter 22 - 76 Jahre)	Effektivität von Tocilizumab bei renaler Amyloidose Tocilizumab + Colchizin (n=11), 1x Umstellung auf Anakinra	Bei 6 Patienten signifikante Reduktion der Proteinurie, bei 10/11 normalisierte Albuminspiegel, bei einem Patienten traten weiterhin Attacken auf.	Tocilizumab scheint bei FMF-assoziierter Amyloidose wirksam zu sein, keine erheblichen unerwünschten Wirkungen.	4
Youngstein T et al. (75)	2017	43 exponierte Kinder während Konzeption bzw. Schwangerschaft Behandlungsgrund der Eltern: 21 CAPS, 6 TRAPS, 6 adulters Still-Syndrom, 5 FMF, 2 Cogan-Syndrome, 1 uncharakteristisches autoinflammatorisches Syndrom	Sicherheit einer IL-1-Blockade während Konzeption, Schwangerschaft und Stillzeit. 8 Mütter unter Canakinumab, 23 Mütter unter Anakinra, 6 Väter unter Canakinumab, 6 Väter unter Anakinra	Unter einer Therapie mit Canakinumab gab es einen Abort in 6. Schwangerschafts-woche, sonst normale Schwangerschaften, Geburten und Kindesentwicklungen. Unter Therapie mit Anakinra ein Abort in 12. Woche, ein Kind mit ektoper Neurohypophyse und unilateraler Nierenagenesie.	Insgesamt beruhigende Daten, jedoch ist dies der zweite dokumentierte Fall von Nieren-agenesie unter Anakinra.	4

Tabelle 2d: Evidenztabelle für sämtliche zitierte kontrollierte Studien (inklusive Abstracts), Studiencharakteristika

Referenz	Jahr	Studien-typ	Patienten-kollektiv	Drop-out Rate	Intervention	Kontrolle	Zielgröße (n)	Ergebnis	Bemerkung	Evidenz
Ben-Zvi I et al. (25)	2017	RCT	25 mit Colchizin-resistentem FMF (alle >18 Jahre)	7 Patienten, alle aus der Placebo-gruppe	12 Anakinra	13 Placebo	Unterschied der Attacken-anzahl; Unterschied der Patienten-zahl mit Attacken-frequenz < 1/Monat	Mittlere Attackenzahl unter Anakinra 1.7±1.7/Monat unter Placebo 3.5±1.9/Monat (p = 0.037)<br>6 Patienten unter Anakinra und kein Patient unter Placebo hatten < 1/ Attacke/Monat (p = 0.005).	Anakinra scheint bei Colchizin-resistentem FMF wirksam zu sein.	1
de Benedetti F et al. (76)	2016	RCT	42 Patienten 19 davon FMF mit Colchizin-Resistenz (Alter unklar)	0	1:1 Randomi-sierung für Canakinumab vs. Placebo	Placebo	Responderrate<br>- Auflösen der Indexattacke nach 15 Tagen<br>- keine weiteren Attacken in 16 Tagen	Erreichen des primären Endpunktes: Colchizin-resistentes FMF: Canakinumab 61.3%, Placebo 6.3% (p < 0.001)	**Abstract**	1b

de Benedetti F et al. (36)	2017	RCT	181, davon 63 FMF-Patienten mit Colchizin-Resistenz	0	1:1 Randomi-sierung für Canakinumab vs. Placebo	Placebo	Assoziation des Genotyps mit dem Ansprechen auf die Therapie bei initial randomisierten Patienten	FMF, Colchizin-Resistenz: kein Unterschied des Ansprechen zwischen Patienten mit homozygoten M694V-Mutationen und anderen Genotypen. MKD: besseres Ansprechen, wenn mindestens eine V377I-Mutation vorliegt. TRAPS: geringes Ansprechen bei Vorliegen der P46L und R92Q-Mutation.	**Abstract**	1b
Kone-Paut I et al. (77)	2017	RCT	181, davon 63 FMF-Patienten mit Colchizin-Resistenz	0	1:1 Randomi-sierung für Canakinumab vs. Placebo	Placebo	Korrelation des AIDAI mit Therapiean-sprechen auf Canakinumab	Der AIDAI korreliert mit unterschied-lichen Aktivitäts-Scores. aber nicht mit CRP und SAA.	**Abstract**	1b
Ozen S et al. (78)	2017	RCT	63 Patienten mit FMF, Colchizin-Resistenz	0	1:1 Randomi-sierung für Canakinumab vs. Placebo	Placebo	- Auflösen der Indexattacke nach 15 Tagen<br>- keine weitere Attacke in 16 Tagen<br>- - keine weiteren Attacken nach 40 Wochen	- keine Attacken nach 16 Wochen: Colchizin-resistentes FMF: Canakinumab 61.3%, Placebo 6.3% ($p < 0.001$)<br>- keine Attacken nach 40 Wochen:<br>Colchizin-resistentes FMF: Canakinumab	**Abstract**	1b

								77.8%, Placebo 30% ($p < 0.051$)		
Ozen, S. et al. (78)	2017	RCT	63 FMF-Patienten mit Colchizin-Resistenz	0	1:1 Randomi-sierung für Canakinumab vs. Placebo	Placebo	Therapiean-sprechen auf Canakinumab	Ansprechen auf Canakinumab vs. Placebo nach 16 Wochen signifikant höher ($p<0.0001$).	**Abstract**	1b
Polat A et al. (79)	2016	RCT	92 Patienten mit FMF (mittleres Alter ca. 8 Jahre)	13 Patienten	42 unter einmal täglich Colchizin	37 unter zweimal täglich Colchizin	Vergleich der Effektivität einer einmalig täglich mit einer zweimal täglichen Colchizin-Gabe. Beurteilung der Krankheits-aktivität mittels modifiziertem Mor-Score und Spiegel der Inflammationsparameter.	Kein Unterschied bzgl. der Sicherheit und Effektivität zwischen den beiden Gruppen.	Colchizin kann 1x/Tag gegeben werden	1b

Tabelle 2e: Evidenztabelle für alle zitierten Übersichtsarbeiten, Studiencharakteristika

**Referenz**	**Jahr**	**Eingeschlossene Studien**	**Ziel**	**Ergebnisse**	**Kommentar**	**Evidenz-stärke**
Erken E et al. (80)	2017			Mehr als 2/3 der Colchizin-resistenten FMF-Patienten sprechen gut auf IL1-Blocker an, daher werden diese empfohlen. Eine kardiale Beteiligung ist selten und ist auf AA-Amyloidose oder chronische systemische Entzündung bezogen.		5
Gul A et al. (81)	2016	unklar	Evaluation des Therapieerfolgs von IL-1-, TNF- und IL-6 R -Blockern	Ca. 2/3 der Patienten mit Colchizin-resistentem FMF sprechen gut auf Anakinra und Canakinumab an, keine Langzeitdaten für Therapie ohne Colchizin-Fortführung, z.B. hinsichtlich der Verhinderung von Amyloidose	Entzündungsmerkmale sowie subjektives Patientenbefinden wurden häufig vernachlässigt. Vorschlag: Patientenfragebogen mit 36 Fragen	5
Haviv R et al. (82)	2016	15 Studien (Fallbeispiele, Serien, Phase 2 und -3 Studien)		Canakinumab ist eine gute Alternative für die meisten Patientenmeisten Patienten mit Colchizin-resistentem FMF. Das Sicherheitsprofil erscheint adäquat.		5
Koga T et al. (83)	2016	unklar	Evaluation des Therapieerfolgs von IL-1-, TNF- und IL-6 R -Blockern	IL-1-, TNF- und IL-6R- Blocker mit vielversprechenden Ergebnissen bei Colchizin-resistentem FMF.		5

Lachmann, HJ et al. (84)	2016			Die Notwendigkeit für validierte Assessments, die verlässlich den therapeutischen Benefit quantifizieren und die Kosten für die Behandlung nach sozioökono-mischen Aspekten rechtfertigen, steigt mit der Verfügbarkeit von neuen Therapien. Die Haupt-probleme bestehen in der exakten Definition von Krankheits-schwere, in der Zusammen-führung von Krankheitsaktivität, Patientenerfahrung, ökonomischer Belastung und krankheitsbezogenen Schäden.	Artikel in Bezug auf Demirkaya et al (MASIF)	5
Ozdogan H et al. (85)	2017	Die Daten von 2 Phase 2-Studien (n=16), einer laufenden randomisierten kontrollierten doppelblinden Phase 3-Studie (n=63), 11 Fallserien (n=35), sowie 2 Abstracts vom ACR-Kongress 2015 (n=45) wurden analysiert.	Die meisten Patienten mit Indikation für eine Biologikatherapie erhielten zunächst Anakinra, v.a. wegen Verfügbarkeit.<br>Der Wechsel zu Canakinumab war hauptsächlich wegen Schmerzen an der Injektionsstelle erfolgt (Hauptnebenwirkung von Anakinra).	Der Wechsel zu Canakinumab nach Anakinra-bedingten Schmerzen an der Injektionsstelle war erfolgreich, Canakinumab Injektionen werden besser vertragen.	Wenige, aber vielversprechende Daten für Canakinumab.	5
Özen S et al. (86)	2017		Pathogenese des FMF und Mangementempfehlungen	Vielverpsrechende anti-IL-1-Therapie bei FMF , Colchizin sollte weitergegeben werden, um eine Amyloidose zu verhindern, da unsicher ist, ob eine anti-IL-1-Therapie diese verhindern kann	Kommentar zu den EULAR-Empfehlungen (23)	5
Ozen S et al. (87)	2017		Definition Colchizin-Resistenz	Frühe Definitionen von Colchizin-Resistenz z.B. 1 Attacke/3 Monate trotz Colchizin 2 mg, aber	Viele Faktoren können zu ungenügendem	5

				manche Patienten tolerieren diese Dosis nicht. Außerdem berücksichtigt es nicht die Schwere von FMF-Manifestationen und Symptomen außerhalb der Fieberattacken wie Myalgien und Vaskulitiden.<br>Außerdem muss die Compliance der Patienten für eine klare Definition miteinbezogen werden.	Ansprechen auf Colchizin führen: mangelnde Compliance, Nebenwirkungen, potentielle Wechsel-wirkungen mit anderen Medikamenten und genetischer Hintergrund.<br>**Kritik an EULAR-Definition**: 11/12 Monaten mit Attacken, wäre bei 1 Attacke/Monat nicht als Colchizin-resistent definiert.	
Sorezi B et al. (88)	2015		Evaluation des Therapieerfolgs von IL-1-, TNF- und IL-6 R -Blockern	IL-1-, TNF- und IL-6R- Blocker mit vielversprechenden Ergebnissen bei Colchizin-resistentem FMF		5
Terreri M et al. (89)	2016	10.341 Artikel, davon wurden 46 genutzt.		Der Gebrauch von IL-1 Rezeptor-antagonisten bei Patienten mit FMF wurde für folgende Situationen beschrieben: Patienten mit inkomplettem Ansprechen auf Colchizin (Colchizin-Resistenz bei 5–10%), oder erhöhten SAA-Spiegeln trotz Therapie, Neben-wirkungen und bei Vaskulitis. Biologika könnten eine wirkungsvolle Alternative zu Colchizin sein.	Wirksamkeit der anti-IL-1-Therapie bei FMF bestätigt.	5

Tabelle 2f: Evidenztabelle für alle zitierten Meta-Analysen und systematischen Reviews, Studiencharakteristika

Referenz	Jahr	Eingeschlossene Studien	Ziel	Ergebnisse	Kommentar	Evidenz-stärke
Demirkaya E et al. (90)	2015	**7** Kontrollierte Studien<br><br>**Patienten**<br>- 57 Colchizin (Dinarello, Goldstein, Zemer, Wright)<br>- 24 ImmunoGard^{TM} (Amaryan)<br>- 22 Interferon-α (Tunca)<br>- 14 Rilonacept (Hashkes)	Evaluation des Therapieerfolgs bei Colchizin, ImmunoGard^{TM}, Rilonacept, Interferon	Reduktion der Attacken durch kontinuierliche Colchizin-Therapie:<br>- $p < 0.001$ (Dinarello)<br>- $p < 0.01$ (Zemer)<br>- $p < 0.002$ (Goldstein)<br>Abbruch der Attacken bei Colchizin-Gabe bei Beginn der Symptome:<br>- unter Colchizin 75%, unter Placebo 10% (Wright)<br>Keine systematische Auswertung der Studien möglich. Keine Berücksichtigung weiterer Studien im Ergebnisteil.	**Bewertung** niedrig	1a
Ozen et al. (18)	2016	**Es wurden systematische Reviews durchgeführt. Diese analysierten verfügbare Evidenz für Wirksamkeit und Sicherheit von Behandlungen in FMF und überprüften die Inzidenz von Komplikationen, einschließlich Amyloidose, Nierenversagen und Unfruchtbarkeit.**	Erstellung von Therapieempfehlungen	Evidenzbasierte Empfehlungen zur Orientierung von Rheumatologen und anderen medizinischen Fachkräften bei der Behandlung und Nachsorge von Patienten mit FMF<br>(siehe 23)	IL-1-Blocker erwähnt als Option bei ungenügendem Ansprechen auf Colchizin **(besonders wichtig bei Patienten mit einer Familienanamnese mit AA-Amyloidose)**, sowie bei **protrahiert verlaufender febrile Myalgie.**	1a

van der Hilst JCH et al. (86)	2016	**27** Kontrollierte Studien, Kohortenstudien, Fallserien und -berichte aus<br>PubMed, Web of Science, und Scopus nach IL-1-Blockern bei FMF, 284 gefunden, 27 benutzt, 24 davon Fallserien, 2 open-label und eine RCT.	Evaluation des Therapieerfolgs bei IL-1-Blockade mit Anakinra (n=64)<br>Canakinumab (n=40)<br>Rilonacept (n=14)	Komplettes Ansprechen unter Anakinra 76.5%, unter Canakinumab 67.5% und unter Rilonacept 14%	Nachweis der Wirksamkeit von IL-1-Blockern bei FMF.	1a
Wu B et al. (85)	2016	**4** Kontrollierte Studien<br><br>**Patienten**<br>- 37 Colchizin (Goldstein, Zemer)<br>- 14 Rilonacept<br>- 24 ImmunoGard™	Evaluation des Therapieerfolgs bei Colchizin, Rilonacept, ImmunoGrad™	Relative Effekte (95% CI) der Interventionen:<br>- Colchizin: 0.21 (0.05 – 0.95) (Goldstein), signifikant;<br>0.78 (0.49 – 1.23) (Zemer), nicht signifikant<br>- Rilonacept: 0.87 (0.59 – 1.26), nicht signifikant<br>ImmunoGradTM:<br>- nicht signifikant für BSG, CRP und Leukozyten	**Bewertung**<br>- Colchizin: niedrig<br>- Rilonacept: moderat<br>- ImmunoGrad™: moderat	1a

Tabelle 3a: Verzerrungsrisiko für sämtliche zitierte Kohortenstudien nach der Newcastle Ottawa Skala (Risiko für Bias)
Insgesamt kann eine Fall-Kontroll- beziehungsweise eine Kohortenstudie neun Sterne erhalten.

Referenzen	Jahr	Selektion	Vergleichbarkeit	Endpunkterfassung
Akar S et al. (33)	2016	+++		++
Barut K et al. (37)	2017	+++		++
Bayram MT et al. (52)	2015	++		++
Bonfrate L et al. (45)	2017	++++	+	+++
Bonyadi MJ et al. (47)	2015	++		++
Cetin P et al. (36)	2015	++		++
Corsia A et al. (56)	2017	++		++
Dermikaya E et al. (61)	2015	++		
Dogan H et al. (48)	2015	+++		+++
Erden A et al. (58)	2016	+++		++
Eroglu FK et al. (39)	2015	+++		++
Herscovici T et al. (50)	2015	++++	+	+++
Knieper M et al. (49)	2017	++		++
Konukbay D et al. (60)	2016	+++		+++
Kucuksahin O et al. (35)	2016	++		++
Laskari K et al. (38)	2017	+++		++
Lofty HM et al. (58)	2016	++		++
Melikoglu MA et al. (55)	2017	++		++

Omma A et al. (57)	2017	++++	+	+++
Özcakar ZB et al. (40)	2014	+++		++
Özdel E et al. (54)	2016	++		+++
Ozen S et al. (34)	2017	++		+
Ozer I et al. (46)	2015	++	+	++
Pecher AC et al. (41)	2017	++		++
Proft, F et al. (44)	2017	+++		++
Rossi-Semerano L et al. (43)	2015	++		++
Stojanovic KS et al. (59)	2016	++		++
Topaloglu R et al. (53)	2016	++		++
Vitale A et al. (42)	2016	++		++
Yesilkaya S et al. (60)	2015	++		++
Yoldas TC et al. (51)	2015	+++		+++

Tabelle 3b: Verzerrungsrisiko für sämtliche zitierte Fall-Kontrollstudien nach der Newcastle Ottawa Skala (Risiko für Bias)
Insgesamt kann eine Fall-Kontroll- beziehungsweise eine Kohortenstudie neun Sterne erhalten.

**Referenzen**	**Jahr**	**Selektion**	**Vergleichbarkeit**	**Endpunkterfassung**
Ben-Zvi I et al. (9)	2015	++++	+	++
Brenner R et al. (30)	2018	++++	+	+
Migita K et al. (32)	2015	++++	+	++
Yalcintepe S et al. (31)	2016	++++	+	++

Suchstrategien (Supplement)

Initiale Suche Medline (PubMed)

#1
familial mediterranean fever[mh] OR familial mediterranean fever[tiab] OR FMF[tiab] OR Colchicine refractory[tiab] OR colchicine-resistant Familial Mediterranean[tiab] OR colchicine-resistant FMF patients[tiab] OR colchicine-resistant Familial Mediterranean patients[tiab]
#2
biological therapy[mh] OR dmards [tiab] OR dmards therapy[tiab] OR Treatment[tiab] OR Colchicine[tiab] OR Colchicine[mh] OR oral colchicine[tiab]
#3
conventional dmard[tiab] OR conventional Disease-modifying antirheumatic drugs[mh] OR conventional Disease-modifying antirheumatic drugs[tiab] OR Antirheumatic Agents[mh] OR Antirheumatic Agents[tiab] OR Anti-Inflammatory Agents, Non-Steroidal[mh] OR Anti-Inflammatory Agents, Non-Steroidal[tiab]
#4
#2 or #3
#5
#1 and #4
#6
#1 and #4: Publication date from 2015/01/01 to 2017/12/31

Dies ergab 249 Treffer.

Zusätzliche Suche Medline (PubMed)

#1
familial mediterranean fever[mh] OR familial mediterranean fever[tiab] OR FMF[tiab] OR Colchicine refractory[tiab] OR colchicine-resistant Familial Mediterranean[tiab] OR colchicine-resistant FMF patients[tiab] OR colchicine-resistant Familial Mediterranean patients[tiab]
#2
biological therapy[mh] OR dmards [tiab] OR dmards therapy[tiab] OR Treatment[tiab] OR Colchicine[tiab] OR Colchicine[mh] OR oral colchicine[tiab]
#3
conventional dmard[tiab] OR conventional Disease-modifying antirheumatic drugs[mh] OR conventional Disease-modifying antirheumatic drugs[tiab] OR Antirheumatic Agents[mh] OR Antirheumatic Agents[tiab] OR Anti-Inflammatory Agents, Non-Steroidal[mh] OR Anti-Inflammatory Agents, Non-Steroidal[tiab]
#4
disease activity[tiab] OR C-Reactive Protein[mh] OR crp level[tiab] OR s100-molecules[tiab] OR Serum Amyloid-A[tiab] OR Amyloidose[tiab] OR AA amyloidosis[tiab] OR erythrocyte sedimentation rate[tiab] OR ESR[tiab] OR serious adverse event[tiab] OR Drug-Related Side Effects and Adverse Reactions[mh] OR SAE reporting[tiab] OR

lactose malabsorption[tiab] OR attacks[tiab] or subclinical inflammation[tiab] OR inflammation[tiab] OR APR[tiab] OR Acute-phase reaction[tiab] OR SAA protein[tiab] OR protein[tiab] OR proteinuria[tiab] OR toxicity[tiab] OR Compliance[tiab] OR Liver enzymes[tiab] OR renal function[tiab] OR glomerular filtration rate[tiab] OR haemodialysis[tiab] OR serious complication[tiab] OR Liver failure[tiab] OR renal failure[tiab] OR drug–drug interactions[tiab] Efficacy[tiab] OR safety[tiab]OR complications[tiab] OR follow-up[tiab] OR sensitivity[tiab] OR specificity[tiab] OR predictive value[tiab] OR acute phase reactants[tiab] OR attack-free period[tiab]

#5
#2 or #3
#6
#1 and #4 and #5
#7
#1 and #4 and #5: Publication date from 2015/01/01 to 2017/12/31

Dies ergab 78 Treffer.

<u>Suche Cochrane Library</u>

#1 MeSH descriptor: [Familial Mediterranean Fever] explode all trees
#2 familial mediterranean fever:ti,ab,kw (Word variations have been searched)
#3 FMF:ti,ab,kw (Word variations have been searched)
#4 Colchicine refractory:ti,ab,kw (Word variations have been searched)
#5 colchicine-resistant Familial Mediterranean:ti,ab,kw (Word variations have been searched)
#6 colchicine-resistant FMF patients:ti,ab,kw (Word variations have been searched)
#7 colchicine-resistant Familial Mediterranean patients:ti,ab,kw (Word variations have been searched)
#8 #1 or #2 or #3 or #4 or #5 or #6 or #7
#9 MeSH descriptor: [Biological Therapy] explode all trees
#10 dmards:ti,ab,kw (Word variations have been searched)
#11 dmards therapy:ti,ab,kw (Word variations have been searched)
#12 Treatment:ti,ab,kw (Word variations have been searched)
#13 Colchicine:ti,ab,kw (Word variations have been searched)
#14 MeSH descriptor: [Colchicine] explode all trees
#15 oral colchicine:ti,ab,kw (Word variations have been searched)
#16 conventional dmard:ti,ab,kw (Word variations have been searched)
#17 MeSH descriptor: [Antirheumatic Agents] explode all trees
#18 conventional Disease-modifying antirheumatic drugs:ti,ab,kw (Word variations have been searched)

#19 Antirheumatic Agents:ti,ab,kw (Word variations have been searched)
#20 MeSH descriptor: [Anti-Inflammatory Agents, Non-Steroidal] explode all trees
#21 Anti-Inflammatory Agents, Non-Steroidal:ti,ab,kw (Word variations have been searched)
#22 #9 or #10 or #11 or #12 or #13 or #14 or #15 or #16 or #17 or #18 or #19 or #20 or #21
#23 #8 and #22 Publication Year from 2015 to 2017

Dies ergab 35 Treffer, nach Dublettenabgleich in EndNote: 33 Treffer
